# Research on On-Line Detection Method of Transformer Winding Deformation Based on VFTO

**DOI:** 10.3390/s21217386

**Published:** 2021-11-06

**Authors:** Yanyun Wang, Guoqiong Zhou, Chunping Zeng, Wenbin Zhang, Yanan Ren, Yi Ke, Hequn Chu, Chunguang Suo

**Affiliations:** 1 College of Science, Kunming University of Science and Technology, Kunming 650504, China; wangyanyun@stu.kust.edu.cn (Y.W.); zgq@kust.edu.cn (G.Z.); zcp@kust.edu.cn (C.Z.); renyanan@stu.kust.edu.cn (Y.R.); chuhequn@kust.edu.cn (H.C.); suochunguang@kust.edu.cn (C.S.); 2 College of Mechanical and Electrical Engineering, Kunming University of Science and Technology, Kunming 650504, China; keyi@stu.kust.edu.cn

**Keywords:** VFTO, frequency response method, winding deformation, equivalent circuit model, online testing

## Abstract

At present, the detection of transformer winding deformation faults is carried out in an offline state, which requires the transformer to cooperate with the implementation of planned power outages, or it takes place after the sudden failure of the transformer when it is out of operation. It is difficult to obtain the status information of the windings online in time. Since the transformer will suffer very fast transient overvoltage (VFTO) impact during operation, combined with the principle of the frequency response method, an online detection method of transformer winding deformation based on VFTO is proposed. In order to study the frequency response characteristics of transformer winding under the impact of VFTO, the generation process of VFTO is simulated by simulation software, and the equivalent circuit model of transformer winding before and after deformation is established. The VFTO signal is injected into the transformer circuit model as an excitation source, and the changes of resonant frequencies of frequency response curve under different deformation types and different deformation degrees of winding are analyzed. The simulation results show that the frequency response curves of different winding deformation types are different. Different deformation degrees are simulated by increasing the radial capacitance by 4%, 13%, and 23%, series inductance by 2%, 4%, and 6%, and longitudinal capacitance by 3%, 6%, and 9%, and the change of resonance frequencies can comprehensively reflect the deformation information of winding. At the same time, the tests of different deformation types and deformation degrees of the simulated winding are carried out. The results show that with the deepening of the change degree of the simulated fault inductance value, the frequency response curve shifts to the low-frequency direction, confirming the feasibility of the online detection method of transformer winding deformation based on VFTO.

## 1. Introduction

In the power system, the transformer is one of the most important pieces of equipment, which is of great significance for the safe operation of the whole power grid. In the system operation, the transformer failure may cause the whole transformer to scrap and shutdown, resulting in large-area power outage. According to incomplete statistics, winding deformation is the main cause of transformer outage and scrap [1,2]. In the long-term operation process, the transformer will be heated and aged, which will reduce the mechanical strength and the ability to withstand electric power of the winding and cause slight deformation of the transformer winding [3]. The accumulation of a slight deformation of transformer winding may lead to an inter-turn short-circuit fault of transformer winding, resulting in serious damage to transformer winding [4]. Therefore, it is of great significance to detect winding deformation before serious winding deformation occurs.

Domestic and foreign scholars have done a lot of research on the detection of transformer winding deformation and formed the following several mature detection methods. The short-circuit impedance method [5,6,7] has been promulgated by the International Electrotechnical Commission (IEC) standards and national standards. By comparing the short-circuit impedance values before and after the transformer winding deformation, it can be judged whether the winding has been deformed or shifted, but the sensitivity to the small deformation of the winding is not high. The online short-circuit impedance method [8] calculates the short-circuit impedance value of the winding by online measuring the power frequency voltage circuit signal of the high and low-voltage sides of the transformer, and then, it judges whether the fault occurs. However, this method is not sensitive to the capacitive winding fault. The low-voltage pulse method [9] judges whether the winding deforms by comparing the change of the response waveform of the winding under the low-voltage pulse excitation. At present, this method is included in the test guidelines of power transformers in IEC and the Institute of Electrical and Electronics Engineers (IEEE), but the test results are easily affected by various electromagnetic interference in the field. The vibration analysis method [10,11] detects online by sensors attached to the oil tank, but short-circuit fault and transient overvoltage will induce high voltage in the transformer shell, which has potential safety hazards for test equipment and testers. The frequency response method [12,13,14] has a small measurement error and simple operation, which is widely used in transformer operation and the production process. However, it is an offline detection method. The state of transformer is different when it is running and offline, which affects measurement and judgment. Reference [15] introduced the online test method of winding deformation based on pulse coupling injection. The excitation signal was injected through the capacitor divider installed on the transformer bushing, but the injected signal may affect the normal operation of the transformer, and other equipment may also be affected.

To sum up, this paper adopts the characteristic that the operation isolation switch in Gas Insulated Substation (GIS) generates a VFTO signal to impact the transformer, and it conducts the research on the online detection of transformer winding deformation based on VFTO. Therefore, this paper analyzes the generation process of VFTO and establishes the concentrated parameter model of winding. By changing different electrical parameters in the equivalent circuit, the axial offset, radial deformation, and inter-cake spacing of transformer winding are simulated. Different deformation degrees are simulated by increasing radial capacitance by 4%, 13%, and 23%, series inductance by 2%, 4%, and 6% and longitudinal capacitance by 3%, 6%, and 9%. The frequency response curves of VFTO-injected normal and fault windings are analyzed, and the transformer fault simulation test is carried out to verify the feasibility of this method.

## 2. Methods and Modeling

### 2.1. Basic Principles

**Figure 1:**
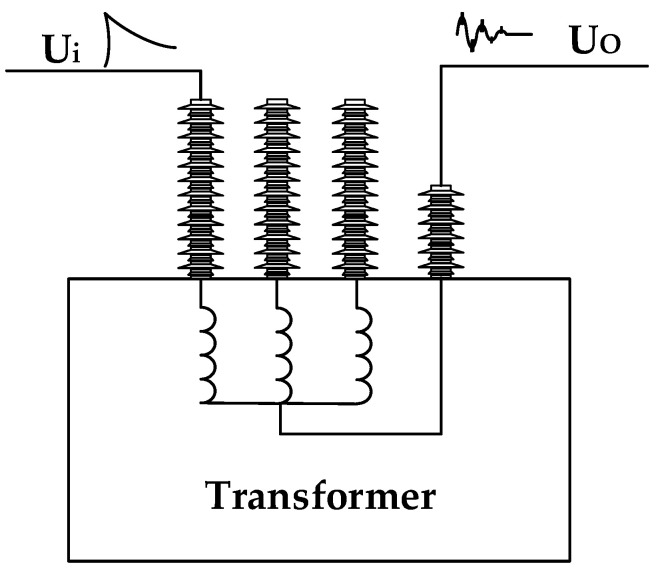
Detection schematic.

Studies at home and abroad have shown that at high frequencies (generally greater than 1 kHz), because the permeability of the core is almost the same as that of air, the influence of the core can be ignored. Therefore, the transformer winding can be regarded as a two-port network composed of capacitance, inductance, and resistance [16,17,18]. The state of the winding determines the parameters of the two-port network. When the state of the winding is different, the parameters will also change. The frequency response curve can describe the transmission characteristics of the two-port network, which is determined by the represented network parameters. Therefore, the winding deformation is detected by combining the frequency response method. Based on the basic idea of frequency response method comparison, three kinds of comparison methods are mainly developed, including comparison in different time periods (vertical comparison), comparison in the same type (horizontal comparison), and comparison between phases (horizontal comparison). The results of comparison in different time periods are the most valuable for fault identification. Therefore, the frequency response curve after the transformer appears is compared with the frequency response curve after a period of operation. As shown in Figure 1, when an excitation voltage signal is applied at one end of the winding, the response signal is obtained at the other end of the winding. The excitation signal and response signal are Fourier transformed by Formulas (1)–(3), and the frequency response curve of the transformer winding is obtained for online detection of winding deformation.

(1) $$U_{i}(f)=f(U_{i}(t))$$

(2) $$U_{o}(f)=f(U_{o}(t))$$

(3) $$TF=20\log_{10}\frac{\left|U_{o}(f)\right|}{\left|U_{i}(f)\right|}$$

where $U_{i}(t)$ and $U_{i}(f)$ are VFTO signals invading the transformer and their spectral functions; $U_{o}(t)$ and $U_{o}(f)$ are the response signal (voltage signal or current signal) output by VFTO after passing through a winding of transformer and its spectrum function; TF is the logarithmic amplitude gain of the frequency response of the winding.

The distributed inductance, capacitance, and resistance parameters of the winding network model are determined by the geometric size and characteristics of the winding and the medium. When a part of the winding encounters mechanical deformation or short-circuit fault, it will cause the change of distribution parameters and finally directly lead to the change of frequency response curve. Therefore, it is theoretically possible to judge whether the winding deforms by comparing the frequency response curves of the normal winding and the measured winding.

### 2.2. Simulation of VFTO Generation Process

**Figure 2:**
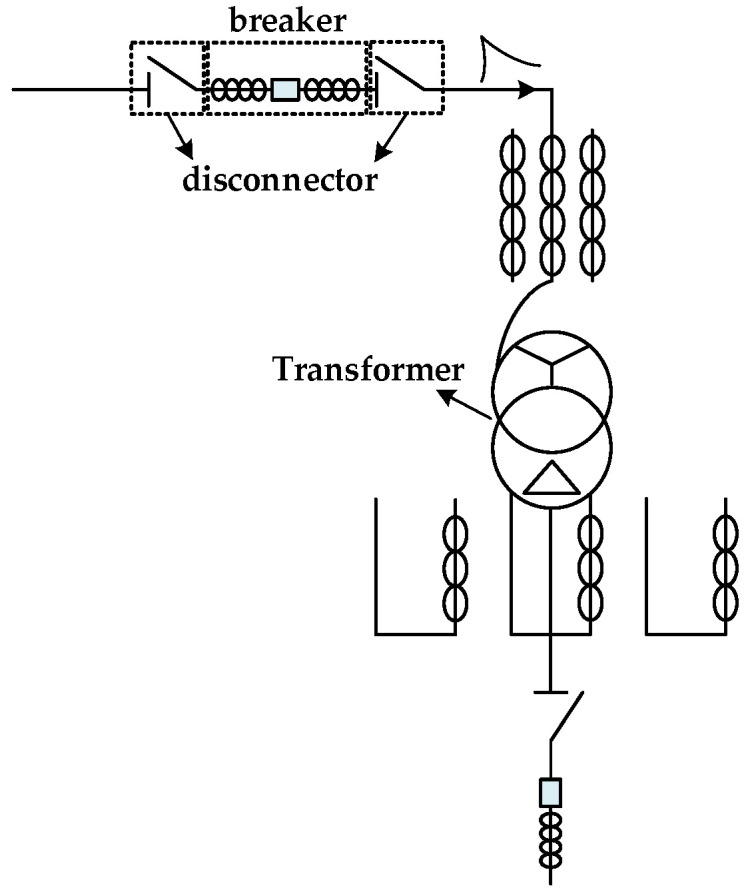
Simplified GIS substation wiring diagram.

In a GIS substation, the fundamental cause of VFTO is that the voltage difference between the two ends of the contact of the disconnector leads to the breakdown of the contact gap. At the moment of the breakdown, the impulse voltage VFTO with an extremely steep rising edge will be generated and propagated inside the GIS [19], which will impact the transformer, as shown in Figure 2. In the closing process of the isolation switch, multiple arc reignitions occur between the contacts. With the decrease of the distance between the contacts, the required breakdown voltage decreases gradually, resulting in the decrease of the average time interval of repeated breakdown and the VFTO amplitude generated by breakdown. Moreover, with the increase of the contact velocity, the number of arc reignitions will decrease. VFTO is an instantaneous overvoltage, usually unipolar and superimposed oscillation, its wavefront time is less than 0.1 μs, the total duration is less than 3 ms, and the oscillation frequency is between 30 kHz and 100 MHz [20], carrying rich spectrum components, so VFTO can be used as an excitation source to detect transformer winding faults.

**Figure 3:**
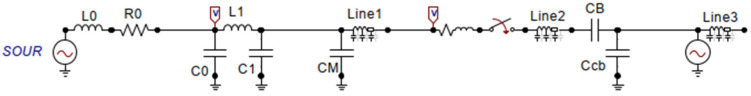
Simulation model of VFTO.

In this section, ATP-EMTP software is used to simulate the closing operation of the disconnector in a 500 kV GIS substation. As shown in Figure 3, the simulation step is 1 ns, and the total simulation time is 30 us. The equivalent model and parameter values of GIS equipment components in the figure are shown in Table 1.

**Figure 4:**
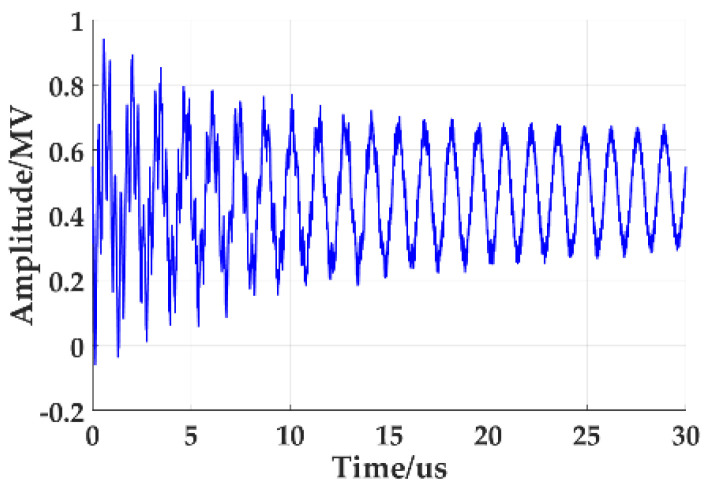
Waveform of VFTO injection transformer front end.

**Figure 5:**
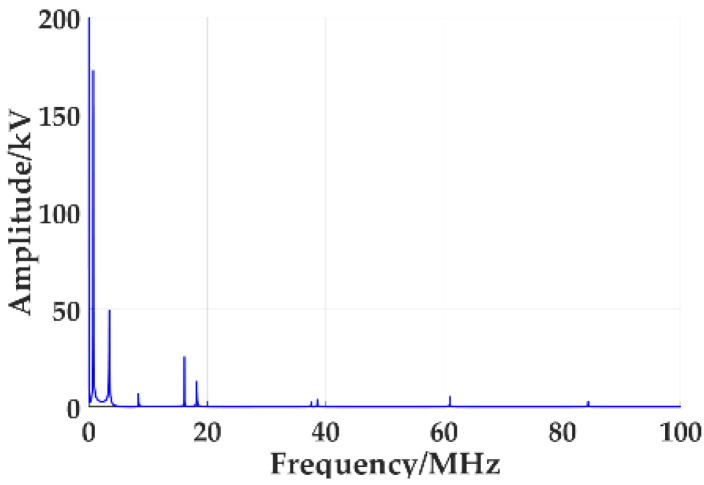
Spectrum of VFTO injected into the front end of the transformer.

The simulation results show that the VFTO waveform at the transformer inlet is shown in Figure 4. At the moment of the disconnector breakdown, the discharge gap is turned on, resulting in the high-frequency oscillation of the voltage in GIS and the generation of VFTO. The VFTO at the transformer entrance is a high-frequency oscillation superimposed on the sine waveform, and its maximum amplitude can reach 1.24 pu. Figure 5 is the frequency spectrum of VFTO waveform. Through analysis, the VFTO waveform has rich frequency components, mainly concentrated in 500 kHz to 20 MHz, as shown in Table 2.

**Figure 6:**
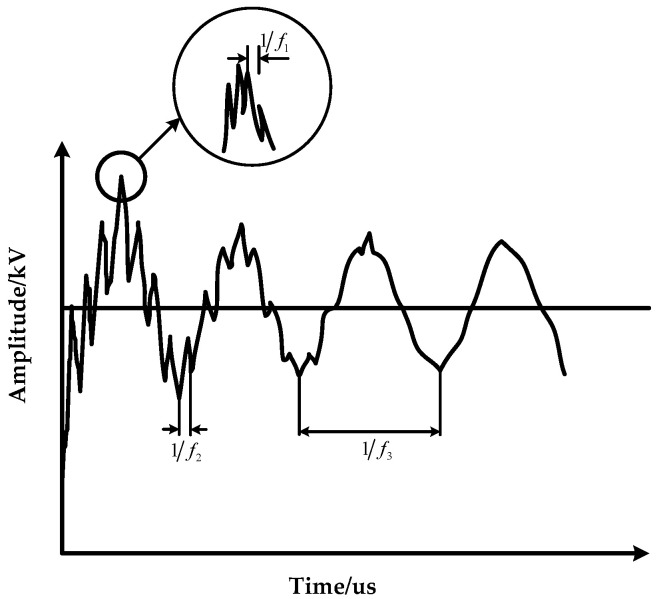
An example of internal steep wave front overvoltage waveform caused by disconnector closing.

An example of the VFTO waveform in the IEC60071-1: 2006 standard is shown in Figure 6. The standard points out that the VFTO waveform usually consists of four components [21,22]:

(1) The amplitude range of the step voltage is 1.0–2.5 times the system voltage;
(2) The $f_{1}$ component in the UHF range, up to 100 MHz;
(3) The $f_{2}$ component in the high-frequency range, up to 30 MHz;
(4) Low-frequency range $f_{3}$ component, range of 0.1–5 MHz.

The comparative analysis of the VFTO waveform in Figure 4 and the standard VFTO waveform shows that the amplitude range of the step voltage is 1.03 times that of the system voltage. $f_{1}=20.4\;\mathrm{MHz}$; $f_{2}=3.52\;\mathrm{MHz}$; $f_{3}=0.7326\;\mathrm{MHz}$; the frequency components of the measured waveform are completely consistent with the four components of VFTO in IEC60071-1: 2006 standard.

### 2.3. Transient Model of Transformer Winding

#### 2.3.1. Establishment of Equivalent Circuit Model of Transformer Winding

**Figure 7:**
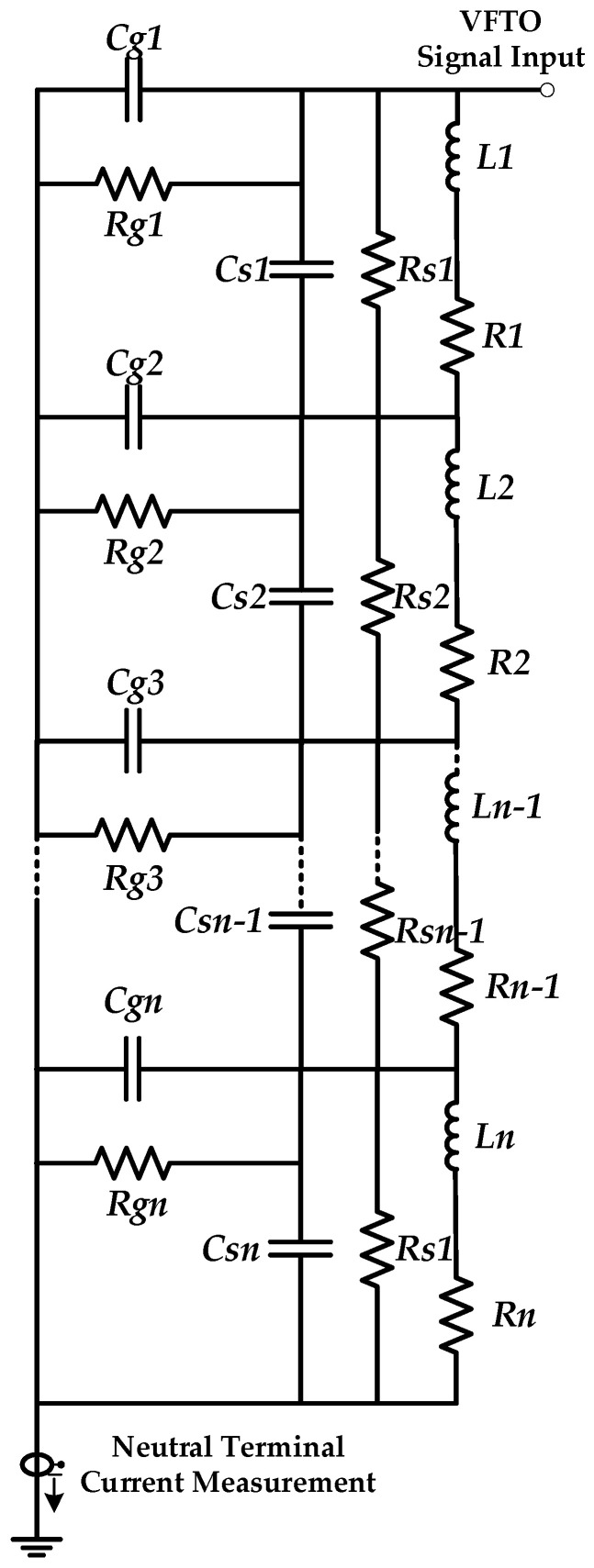
Equivalent circuit model of transformer winding.

At present, there are many studies on the transient circuit model of transformer winding at high frequency, mainly including the multi-conductor transmission line model that takes each turn of transformer winding as a transmission line, the uniform transmission line model that combines single-conductor and multi-conductor transmission lines, and the mixed model of a multi-conductor transmission line and concentrated parameter [23,24,25,26]. However, the calculation of a multi-conductor transmission line model is large, and the error of the uniform transmission line model is large. It is difficult to determine the parameters of components in the hybrid model. In Reference [27], ANSYS Maxwell was used to establish the two-dimensional model of the transformer, and the inter-turn capacitance, inter-cake capacitance, radial capacitance, and inductance of the transformer winding were calculated. Then, the equivalent circuit model of the winding was established. By referring to this model, the transformer winding is equivalent to a two-port network composed of capacitance, inductance, and resistance [28], as shown in Figure 7.

The high-voltage side winding of the transformer is composed of 14 windings, and each disc unit includes series inductance L, series resistance R, longitudinal capacitance Cs, longitudinal resistance Rs, radial capacitance Cg, and radial resistance Rg. Series inductance L represents the inductance of winding. Series resistance R is the resistance of winding. The longitudinal capacitance Cs is composed of inter-turn capacitance and inter-cake capacitance. Longitudinal resistance Rs is the dielectric loss between winding turns or cakes. Radial capacitance Cg consists of capacitance between winding and core, capacitance between winding and winding, and capacitance between winding and tank. Radial resistance Rg is the dielectric loss between winding turns or cakes. The VFTO signal at the transformer winding inlet and the response signal at the output end are measured to calculate the frequency response curve.

#### 2.3.2. Parameter Calculation of Transformer Winding Equivalent Circuit

1. Calculation of series resistance parameters

The calculation formula of resistance per unit length of transformer winding turns is as follows:

(4) $$R=\sqrt{\pi f\mu \rho }/2(a+b).$$

In the formula, f is the calculated frequency. μ is the permeability of a conductor. ρ is the resistivity of the conductor. a and b are the two side lengths of the conductor’s rectangular section.

2. Calculation of series inductance parameters

The frequency of VFTO is high. After the transformer is injected, the transformer core has little effect on the transmission of energy, so it can be ignored [12,13,14]. Therefore, the winding of the transformer is equivalent to a hollow coil. The calculation formula of inductance of a hollow coil can be used to calculate inductance of winding. Since the average diameter of the winding is much larger than the thickness of the wire cake, the inductance of the winding is calculated by using the formula of the inductance calculated by the planar coil. The formula is as follows:

(5) $$L=\frac{\mu_{0}}{8\pi }D_{m}\psi n^{2}$$

in the formula, $\mu_{0}$ is the vacuum permeability. n is the number of turns of single-line cake. $D_{m}$ is the average diameter of winding. ψ is the correlation function.

3 Calculation of Longitudinal Capacitance Parameters

The longitudinal capacitance includes inter-cake capacitance and inter-turn capacitance. Since the average diameter of the transformer coil is much larger than the width of the coil, the inter-turn capacitance $C_{T}$ and inter-cake $C_{DA}$ capacitance can be obtained according to the calculation principle of the plate capacitance:

(6) $$C_{T}=\frac{\varepsilon_{0}\varepsilon_{p}\times \pi D_{m}(w+t_{p})}{t_{p}}.$$

In the formula, w is the bare width of the axial wire. $t_{p}$ is the total thickness of paper insulation. $\varepsilon_{0}$ is the dielectric constant of vacuum. $\varepsilon_{p}$ is the relative dielectric constant of paper insulation.

(7) $$C_{DA}=\frac{\varepsilon_{0}\varepsilon_{de}\pi D_{m}B}{t_{d}}$$

In the formula, $\varepsilon_{de}$ is the equivalent dielectric constant of the inter-cake insulation. B is the width of the line cake. $t_{d}$ is the insulation thickness between cakes.

4 Calculation of radial capacitance parameters

The radial capacitance Cg is composed of the capacitance between the winding and the core, the capacitance between the winding and the winding, and the capacitance between the winding and the tank. The calculation formula is as follows:

(8) $$C_{g}=\frac{2\pi H\varepsilon_{0}\varepsilon_{we}}{\ln\frac{R_{w}}{R_{i}}}.$$

In the formula, $\varepsilon_{we}$ is the equivalent dielectric constant of winding and core medium. H is the axial height of winding. $R_{w}$ is the inner radius of winding. $R_{i}$ is the outer radius of the core.

**Figure 8:**
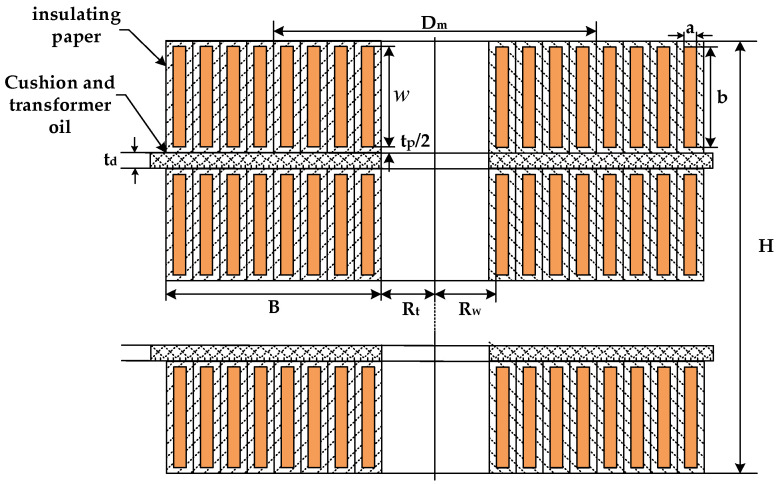
Insulation diagram between cakes.

The schematic diagram of the insulation between the winding pie of the transformer is shown in Figure 8, and the calculation parameters in Formulas (4)–(8) are marked in the figure.

The relevant size parameters of a 500 kV transformer are shown in Table 3. According to these parameters and formulas, the equivalent circuit parameters of transformer winding are calculated, as shown in Table 4.

The change of transformer winding equivalent circuit parameters will affect its frequency response characteristics. Since the winding is composed of inductance, capacitance, and resistance, the phenomenon of multi-frequency resonance will occur in the transformer winding. The peak on the frequency response curve is due to the series resonance inside the transformer winding. The valley on the frequency response curve is due to the parallel resonance inside the transformer winding. For the determined transformer winding, the corresponding equivalent circuit parameters are constant; then, the frequency response curve is also uniquely determined. However, when the transformer winding deforms, the corresponding equivalent circuit parameters will change, which is reflected in the change of frequency or amplitude of the peak and trough on the frequency response curve. Therefore, the deformation type and degree of transformer winding can be analyzed through the change of the frequency response curve.

**Figure 9:**
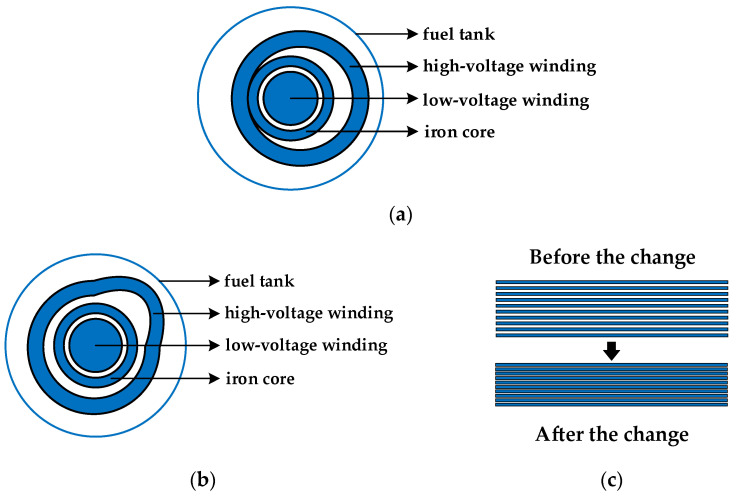
Fault diagram of axial offset, radial deformation and inter-cake spacing of winding: (**a**) Axial offset diagram of winding; (**b**) Radial deformation diagram of winding; (**c**) The change of inter-cake spacing.

In Reference [25], the variation of the equivalent circuit parameters of the winding corresponding to the faults of the winding axis deviation, radial deformation, and the change of inter-cake spacing is simulated, as shown in Table 5. The fault diagram of winding axial offset, radial deformation, and disc spacing change is shown in Figure 9. When the axial offset fault occurs in the high voltage winding, the capacitance between the high-voltage and low-voltage winding changes greatly. Since the distance between the high-voltage winding and the oil tank is far, the capacitance between the two changes little. Overall, the radial capacitance of the winding increases. When the radial deformation of the high voltage winding occurs, the winding will produce a plum blossom or drum-shaped deformation phenomenon. The series inductance and longitudinal capacitance increase with the deepening of the deformation degree. Since the capacitance between the high-voltage winding and the low-voltage winding decreases, and the capacitance between the tank increases, the two offset each other, so the radial capacitance value does not necessarily decrease with the increase of the deformation variable. When the transformer winding is subjected to axial electromagnetic force, the spacing between the winding wire cakes decreases, resulting in an increase in the capacitance between the cakes. Overall, the longitudinal capacitance increases.

## 3. Simulation and Experimental Results

### 3.1. Simulation Study on Winding Deformation

In the previous section, the VFTO simulation model and the equivalent circuit model of transformer winding are studied, and the changes of equivalent circuit parameters caused by the axial offset, radial deformation of winding, and the change of inter-cake spacing are analyzed. In this section, the joint circuit model will be established in ATP-EMTP software for simulation to study the variation characteristics of frequency response curves of windings under different deformation types and degrees. According to Figure 6, the equivalent circuit model of transformer is established in ATP-EMTP software, and the VFTO waveform generated by Figure 3 is used as the excitation source. By changing the parameters of transformer winding, an equivalent circuit can correspond to different winding deformation types and deformation degrees.

#### 3.1.1. Simulation of Different Fault Types

**Figure 10:**
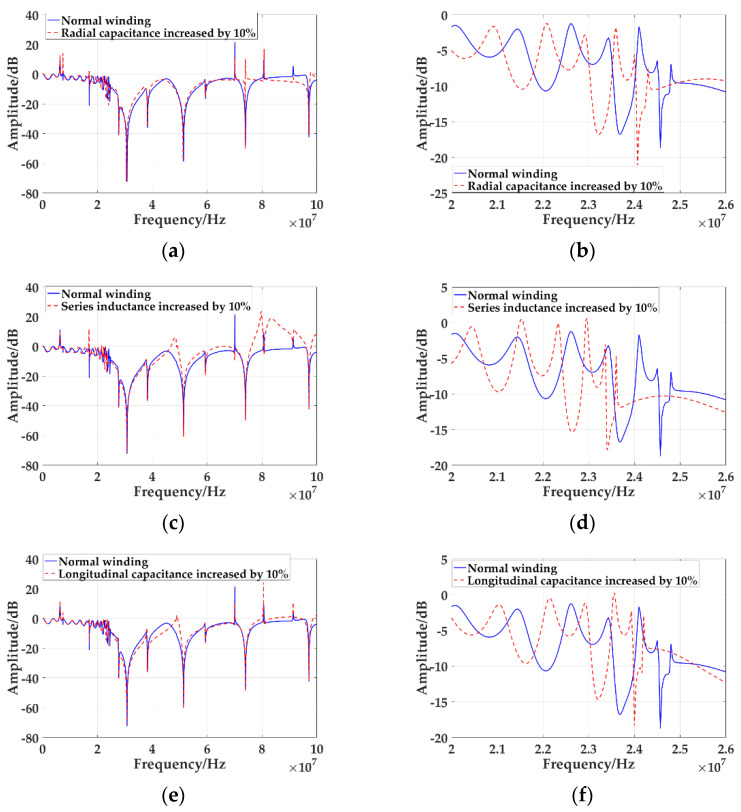
Simulation results of three types of winding faults: (**a**) Frequency response curve of overall winding axis offset; (**b**) Frequency response curves of winding radial deformation from 20 MHz to 26 MHz; (**c**) Frequency response curve of the whole winding radial deformation; (**d**) Frequency response curve of radial winding deformation 20 MHz to 26 MHz; (**e**) The overall frequency response curve of winding inter-cake spacing variation; (**f**) Frequency response curve of winding inter-cake spacing variation 20 MHz to 26 MHz.

The faults of winding axis offset, radial deformation, and inter-cake spacing change are simulated respectively, and the input voltage and output voltage are measured. According to Formula (3), the frequency response curves of three fault types are calculated, as shown in Figure 10. Among them, the horizontal axis represents the frequency. Since the frequency of VFTO can reach 100 MHz, the range of the horizontal axis is set to 100 MHz. The longitudinal axis represents the amplitude. The solid line represents the frequency response curve of the normal winding, and the dashed line represents the frequency response curve of the fault winding.

Figure 10a shows the comparison of the frequency response curve of the radial capacitance increase by 10% due to the axis deviation of the winding with that of the normal winding, and a sharp peak appears at 7.47 MHz and 73.96 MHz. The curve is overall forward after 7.47 MHz. Figure 10b is the frequency response curve of winding radial deformation A to S. Compared with the normal winding frequency response curve, the curve moves down as a whole and toward the low-frequency direction. Figure 10c shows the frequency response curve of the series inductance increase by 10% due to the radial deformation of the winding. Compared with the frequency response curve of the normal winding, there is a sharp peak at 16.93 MHz, and the peak at 80.66 MHz becomes a trough, and there is a peak before and after. Figure 10d shows the frequency response curves of winding radial deformation 20 MHz to 26 MHz. It can be seen from the figure that compared with the frequency response curve of normal winding, the curve moves to the low-frequency direction as a whole. Figure 10e shows the overall frequency response curve of the increase of longitudinal capacitance by 10% due to the change of inter-cake spacing of the windings. Compared with the frequency response curve of the normal winding, there is a sharp peak at 7.466 MHz and 80.6 MHz, and the curve moves forward after 10.53 MHz. Figure 10f is the frequency response curve of the change of inter-cake spacing of the windings from A to S. It can be seen from the figure that compared with the frequency response curve of normal winding, the curve moves to the low-frequency direction and upwards.

#### 3.1.2. Simulation of Different Fault Levels

By increasing or decreasing the percentage of series inductance, radial capacitance, and longitudinal capacitance value, the fault degree of three deformation types of winding axial offset, radial deformation, and inter-cake spacing change is simulated. By comparing the frequency response curves of normal winding and different degrees of deformation, the degree of winding deformation can be effectively diagnosed by analyzing the difference between the curves.

1. Axial offset of winding

**Figure 11:**
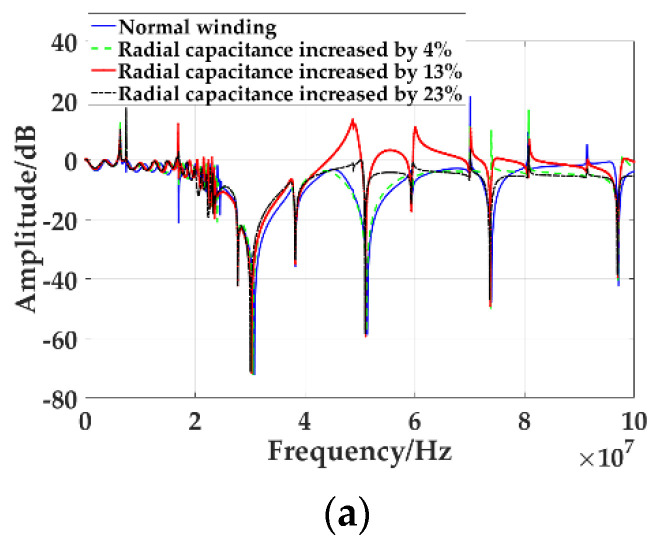

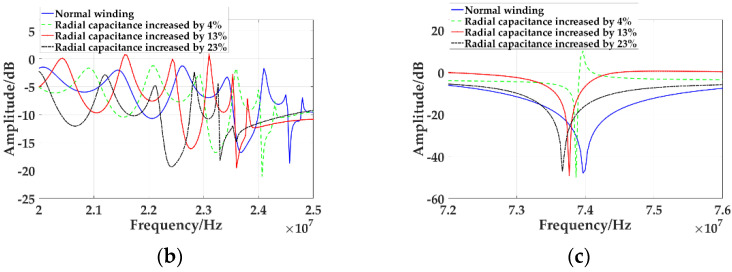
Frequency response curves of different degrees of axial offset of winding: (**a**) Frequency response curve from 0 MHz to 100 MHz; (**b**) Frequency response curve from 20 MHz to 25 MHz; (**c**) Frequency response curve from 72 MHz to 76 MHz.

With the increase of winding axis offset, the radial capacitance will also increase. By increasing the percentage of radial capacitance to simulate the degree of axial offset of winding, the frequency response curves of different axial offset of winding are compared, as shown in Figure 11.

The frequency response curves of the simulated radial capacitance increase by 4%, 13%, and 23%, which are compared with those of the normal winding. As shown in Figure 11a, the transverse axis is the frequency, ranging from 0 MHz to 100 MHz, and the longitudinal axis is the amplitude. Figure 11b is the frequency response curve from 20 MHz to 25 MHz in the middle-frequency band, and Figure 11c is the frequency response curve from 72 MHz to 76 MHz in the high-frequency band. It can be seen that the frequency of the trough moves to the low frequency as the axial offset increases, and the greater the axial offset is, the greater the movement. The frequency and amplitude of the trough in the frequency response curve change with the axial offset, as shown in Table 6. With the increase of the axial offset, the frequency of the trough in the frequency response curve decreases. The movement of the mid-band trough frequency is greater than that of the high band, and the amplitude is basically not affected by the deformation.

2 Radial deformation of winding

**Figure 12:**
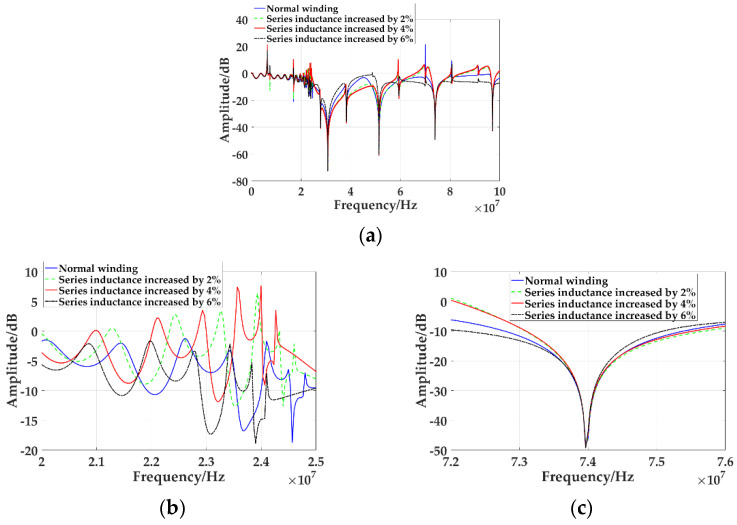
Frequency response curves of different degrees of radial deformation of winding: (**a**) Frequency response curve from 0 MHz to 100 MHz; (**b**) Frequency response curve from 20 MHz to 25 MHz; (**c**) Frequency response curve from 72 MHz to 76 MHz.

The increase of radial deformation of high voltage winding leads to the increase of longitudinal capacitance and series inductance. The slight radial deformation of winding is simulated by changing the increment of series inductance. The frequency response curves of normal winding and series inductance increased by 2%, 4%, and 6% are simulated, as shown in Figure 12a.

Figure 12b is the frequency response curve of different degrees of radial deformation from 20 MHz to 25 MHz. It can be seen that with the increase of radial deformation, the curve moves to the low-frequency direction, and the greater the deformation, the greater the amount of movement. Figure 12c is the trough of different degrees of radial deformation between 72 MHz and 76 MHz. It can be seen that the frequency and amplitude of the curve are basically unchanged at higher frequencies. The changes of the frequency and amplitude of the trough in the frequency response curve are shown in Table 7. It can be seen that with the increase of the radial deformation degree, the frequency value of the trough decreases, and the radial deformation of the winding has a great influence on the frequency and amplitude of the trough at lower frequencies, but it has little effect on the frequency and amplitude of the trough at higher frequencies.

3. Change of inter-cake spacing

**Figure 13:**
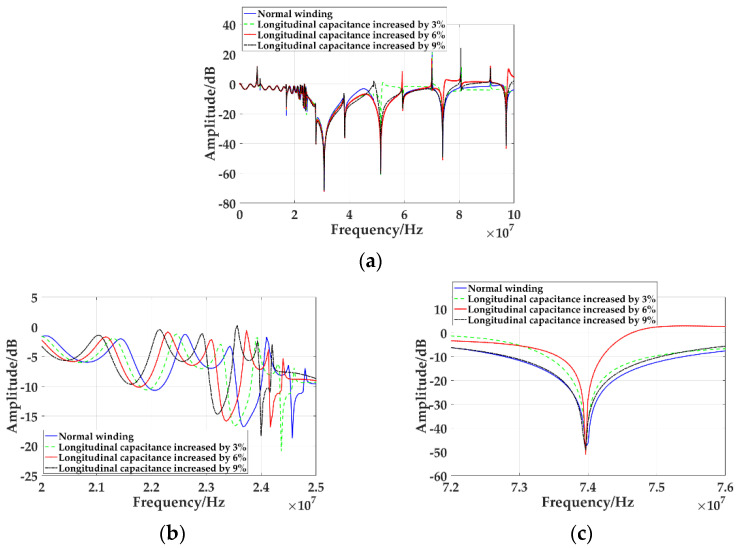
Frequency response curves of different winding inter-cake spacing: (**a**) Frequency response curve from 0 MHz to 100 MHz; (**b**) Frequency response curve from 20 MHz to 25 MHz; (**c**) Frequency response curve from 72 MHz to 76 MHz.

With the decrease of the distance between winding discs, the longitudinal capacitance increases continuously. By changing the increment of the longitudinal capacitance, the change of the winding inter-cake spacing is simulated. The frequency response curves of simulated normal winding and longitudinal capacitance increase by 3%, 6%, and 9%, as shown in Figure 13a.

Figure 13b shows the frequency response curves of different cake spacing from to 25 MHz. It can be seen that with the decrease of cake spacing, the frequency response curve moves to the lower frequency direction, and the larger the deformation is, the greater the amount of movement. Figure 13c shows the troughs of different inter-cake spacings between 72 MHz and 76 MHz. It can be seen that in the high-frequency part, with the decrease of inter-cake spacing, the frequency and amplitude of the troughs do not change significantly. Changes in the frequency and amplitude of the trough of the frequency response curve are shown in Table 8. It can be seen that before 30.83 MHz, the frequency of the trough decreases and moves upward with the decrease of the spacing between the winding panes. The change of winding pancake spacing has a great influence on the frequency and amplitude of the trough at lower frequency, but it has no influence on the frequency and amplitude of the trough at higher frequency.

### 3.2. Confirmatory Test

**Figure 14:**
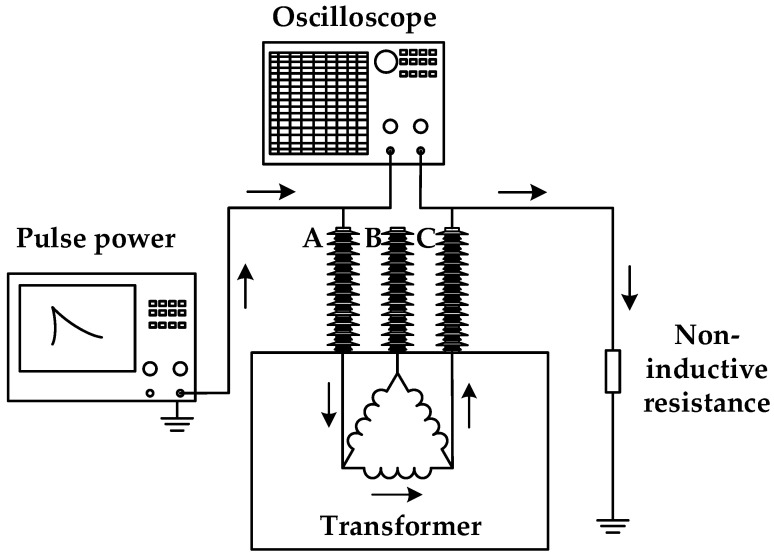
Experimental wiring diagram.

In order to verify the feasibility of the detection method proposed in this paper, an experimental platform is set up on the 10 kV transformer in the laboratory for offline test. The offline test mainly verifies the feasibility of the detection method. The model of the transformer is S11-M-80/10, the rated capacity is 80 kVA, and the rated voltage is 10,000/400 V. Taking into account the cost of field experiments, the simulation of VFTO power supply developed by our laboratory for high-voltage second pulse voltage generator, using a double exponential pulse signal, so that the experimental process is simple and controllable, and has a certain equivalence. The generator can output a double exponential pulse signal with amplitude 0 – 2 kV and rise time <5 ns, and a large number of winding experiments can be carried out. The experimental wiring diagram is shown in Figure 14. The excitation voltage signal generated by the pulse source is injected into the high voltage A-phase winding, and the response signal is output from the high voltage C-phase winding. The excitation signal and response signal are collected by oscilloscope, and the frequency response curve of the normal winding is obtained. Among them, the oscilloscope uses Tektronix MSO3024, the bandwidth is DC-200MHz, and the sampling rate is 2.5 GS/s for a 4-channel 8-bit digital oscilloscope. During the test, the pulse amplitude at the first end of the injection winding is 2 kV, the frequency is 50 Hz, and the pulse width is 200 ns.

**Figure 15:**
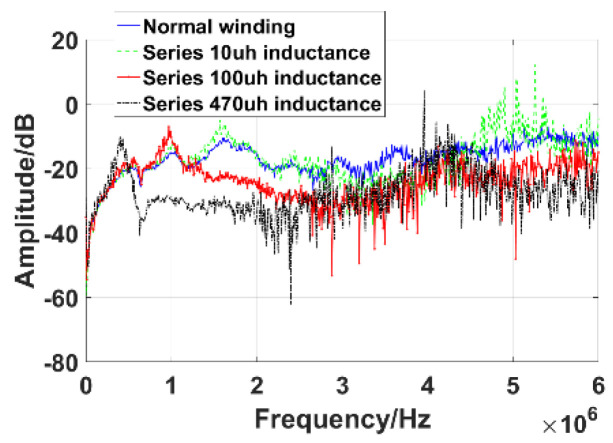
Experimental results.

In order not to damage the transformer, the inductive fault of winding is simulated by series inductances with inductance values of 10 μH, 100 μH, and 470 μH at the inlet end of high-voltage bushing of transformer 10 kV. The parameters of equivalent circuit model of winding are changed by series inductances, and then, the frequency response curve is changed, as shown in Figure 15. It can be seen that with the increase of series inductance, the main peak of the curve shifts to the low-frequency direction, which confirms that the method can be used to detect transformer winding faults.

## 4. Discussion

(1) In this paper, an online detection method for transformer winding deformation based on VFTO is proposed. The VFTO signal at the transformer entrance of a 500 kV GIS substation is simulated by ATP-EMTP software. The equivalent circuit model of the transformer is calculated by formula, and the changes of equivalent circuit parameters caused by the faults of axial offset, radial deformation, and changing of inter-cake spacing are analyzed.
(2) In this paper, ATP-EMTP software is used to establish the circuit model of VFTO impact transformer winding with different fault types and fault degrees, and the overall characteristics of the frequency response curve and the change of the trough are obtained. The frequency response curves of different fault types reflect certain differences. Different deformation degrees do not change the overall characteristics of the frequency response curve. However, with the deepening of deformation degree, the displacement of the curve in the low-frequency direction increases, and it has a great influence on the frequency and amplitude of the lower frequency trough, but it has little influence on the frequency and amplitude of the higher frequency trough. At the same time, because the equivalent circuit model of transformer winding is universal, this result is applicable to the fault analysis of different transformers.
(3) In this paper, an experimental platform is built to show that the frequency response curve shifts to the low-frequency direction with the deepening of the change of inductance value, which verifies the feasibility of this method.
(4) This paper will study the broadband voltage sensor, collecting the VFTO signal and response signal. A large number of fault winding experiments are carried out to study the fault identification method.

## Figures and Tables

**Table 1:** Equivalent model and parameter values of GIS equipment components.

Components	Equivalent Model	Parameter Values
transformer	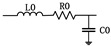	$\begin{array}{l} \mathrm{L0}=176\;\mathrm{mH}\\ \mathrm{R0}=1\;\Omega \\ \mathrm{C0}=5000\;\mathrm{pF} \end{array}$
transformer bushings	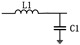	$\begin{array}{l} \mathrm{L1}=0.03\;\mathrm{mH}\\ \mathrm{C1}=320\;\mathrm{pF} \end{array}$
lightning arrester	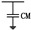	CM=19 pF
electric arc	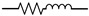	$\begin{array}{l} L=0.25\;\mu H\\ R=2\;\Omega \end{array}$
breaker	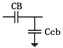	$\begin{array}{l} \mathrm{CB}=540\;\mathrm{pF}\\ \mathrm{Ccb}=240\;\mathrm{pF} \end{array}$

**Table 2:** Main parameters of VFTO spectrum.

Parameters	First Resonance Point	Second Resonance Point	Third Resonance Point	Fourth Resonance Point	Fifth Resonance Point
frequency (MHz)	0.733	3.467	8.4	16.17	18.23
amplitude (kV)	172.9	49.68	6.929	25.95	13.2

**Table 3:** Structural dimension parameters of high voltage windings.

Parameter	Parameter Value
Number of cakes	96
Number of pads × wide	16 × 50 mm
Net metal height of conductor	9.65 mm
Internal radius of high-voltage coil	1074 mm
External radius of inner winding	1011 mm
Radial height of wire cake	348 mm
Average diameter of wire cake	1245 mm
Axial height of coil	2000 mm
Outer radius of core	600 mm
Outside radius of high voltage coil	174 mm

**Table 4:** Parameters of transformer winding equivalent circuit.

Parameter	L	R	Cs	Rs	Cg	Rg
Winding equivalent parameters	0.6805 μH	5 mΩ	30.15 pF	1.06 MΩ	0.12 nF	32 MΩ

**Table 5:** Changes of winding deformation corresponding parameters.

Winding Fault Type	Corresponding Parameter Changes
Axial offset	Radial capacitance increases
Radial deformation	Longitudinal capacitance and inductance increase with increasing deformation
Changes of cake spacing	The decrease of cake spacing leads to the increase of longitudinal capacitance

**Table 6:** Influence of different axis deviation degree of winding on frequency and amplitude of wave trough.

Trough	Characteristic Quantity	Normal	Offset 4%	Offset 13%	Offset 23%
First trough	Frequency/MHz	97.13	97.06	96.96	96.9
	Amplitude/dB	−42.56	−40.52	−39.5	−38.65
Second trough	Frequency/MHz	73.96	73.86	73.76	73.66
	Amplitude/dB	−48.04	−50.11	−49.07	−47.24
Third trough	Frequency/MHz	51.4	51.23	51.1	50.96
	Amplitude/dB	−58.82	−57.94	−59.34	−58.48
Fourth trough	Frequency/MHz	30.83	30.57	30.33	30.1
	Amplitude/dB	−72.45	−72.26	−71.61	−71.27
Fifth trough	Frequency/MHz	24.57	24.07	23.6	23.3
	Amplitude/dB	−18.75	−21.08	−19.5	−18.25
Sixth trough	Frequency/MHz	24.3	23.8	23.37	23.07
	Amplitude/dB	−7.619	−9.035	−9.585	−10.74

**Table 7:** Influence of winding radial deformation on frequency and amplitude of wave trough.

Trough	Characteristic Quantity	Normal	Deformation 2%	Deformation 4%	Deformation 6%
First trough	Frequency/MHz	51.4	51.4	51.4	51.4
	Amplitude/dB	−58.82	−59.75	−60.82	−61.05
Second trough	Frequency/MHz	30.83	30.83	30.8	30.77
	Amplitude/dB	−72.45	−71.19	−70.55	−72.86
Third trough	Frequency/MHz	24.57	24.4	24.07	23.9
	Amplitude/dB	−18.75	−12.62	−9.032	−18.91
Fourth trough	Frequency/MHz	24.3	24.17	23.8	23.67
	Amplitude/dB	−7.619	−3.473	−1.512	−10.11
Fifth trough	Frequency/MHz	23.67	23.5	23.2	23.07
	Amplitude/dB	−16.77	−12.55	−11.86	−17.31
Sixth trough	Frequency/MHz	23.1	22.87	22.53	22.43
	Amplitude/dB	−6.977	−4.252	−4.488	−8.409

**Table 8:** Influence of different inter-cake spacings of winding on frequency and amplitude of wave trough.

Trough	Characteristic Quantity	Normal	Deformation 3%	Deformation 6%	Deformation 9%
First trough	Frequency/MHz	73.96	73.96	73.96	73.96
	Amplitude/dB	−48.04	−50.64	−50.97	−49.3
Second trough	Frequency/MHz	51.4	51.4	51.4	51.36
	Amplitude/dB	−58.82	−61.08	−58.11	−60.58
Third trough	Frequency/MHz	30.83	30.8	30.77	30.73
	Amplitude/dB	−72.45	−72.32	−71.96	−71.28
Fourth trough	Frequency/MHz	24.57	24.37	24.17	24
	Amplitude/dB	−18.75	−20.89	−16.81	−18.33
Fifth trough	Frequency/MHz	24.3	24.17	23.97	23.77
	Amplitude/dB	−7.619	−8.077	−6.981	−5.688
Sixth trough	Frequency/MHz	23.67	23.5	23.37	23.2
	Amplitude/dB	−16.77	−16.56	−15.84	−14.71

## Data Availability

Link: https://pan.baidu.com/s/12CKU9IVqkkVvrApTgqEwHQ (accessed on 1 November 2021). Extraction codes: 5fpj.

## References

[1] Liu Y., He S., Sun L., Xie Y., Zhao S., Qin J. Transformer winding deformation detection analysis and typical cases. Proceedings of the 2016 IEEE International Conference on High Voltage Engineering and Application (ICHVE).

[2] Li Z., Hao Z., Yan C., Dang Y., Xu H., Zhang B. Deformation simulation and analysis of power transformer windings. Proceedings of the 2016 IEEE PES Asia-Pacific Power and Energy Engineering Conference (APPEEC).

[3] Zhang H., Zhang H., Ma Q., Liu Y., Wang S. (2019). Analysis on elastic and plastic deformations of power transformer winding based on finite element method. High Volt. Apparatus..

[4] Tang Z., Peng M., Li G., Wan X., Liu R. (2018). Diagnosis of inter-turn short circuit fault of transformer winding based on repetitive surge oscillograph. Electr. Power Autom. Equip..

[5] Ye Z., Yu W., Gou J., Tan K., Zeng W., An B., Li Y. (2020). A calculation method to adjust the short-circuit impedance of a transformer. IEEE Access.

[6] Wang X., Li Y., Li L. (2019). Design and calculation analysis for high impedance transformer based on field-circuit coupled method. High Volt. Appar..

[7] Huang Z., Chen Y.H., Shi S.M., Luo L.F. (2019). Study on characteristic parameters of short-circuit impedance for a four-winding inductive filtering transformer in power system supplying nonlinear loads. IEEE Access.

[8] Wu Y., Gu L., Zhang X., Wang J. (2019). An on-line identification method for short-circuit impedance of transformer winding based on sudden short circuit test. Lect. Notes Electr. Eng..

[9] Li H., Huang D., Zhang B., Chen N. (2020). Research in detection of winding transformer variation based on improved LVI method. Nanjing Univ. Sci. Technol..

[10] Yu Z., Li D., Chen L. Statistical analysis of vibration characteristics of power transformers with different voltage levels. Proceedings of the IEEE International Conference on Properties and Applications of Dielectric Materials.

[11] Monteiro C.S., Rodrigues A.V., Viveiros D., Linhares C., Mendes H., Silva S.O., Marques P.V.S., Tavares S.M.O., Frazão O. (2021). Optical fiber sensors for structural monitoring in power transformers. Sensors.

[12] Kuniewski M. (2020). FRA diagnostics measurement of winding deformation in model single-phase transformers made with silicon-steel, amorphous and nanocrystalline magnetic cores. Energies.

[13] Kornatowski E., Banaszak S. (2020). Frequency response quality index for assessing the mechanical condition of transformer windings. Energies.

[14] Alsuhaibani S., Khan Y., Beroual A., Malik N.H. (2016). A review of frequency response analysis methods for power transformer diagnostics. Energies.

[15] Ding G., Chen Q., Tian Y., Tian Y., Liu Y., Yang H., Li C. (2017). Simulation and experiment study on the validity of the method for online detecting slight deformation of transformer winding based on the nanosecond pulse response method. High Volt. Appar..

[16] Gawrylczyk K., Banaszak S., Ilinca A. (2021). Recent developments in the modelling of transformer windings. Energies.

[17] Mao C., Wu J., Duan W., Han Y., Zhang L., Liu Q., Wang S., Wang S., Zhang H., Wang S. (2019). Research of corresponding relationship between transformer winding deformation and variation of equivalent electrical parameters. High Volt. Appar..

[18] Pham D.A., Gockenbach E. (2016). Analysis of physical transformer circuits for frequency response interpretation and mechanical failure diagnosis. IEEE Trans. Dielectr. Electr. Insul..

[19] Chen W., Zhang W., Wang L., Li C., Qiao Y. (2021). Research and experiments on an external miniaturized VFTO measurement system. Electr. Technol..

[20] Ma G.M., Li C.R., Li X., Zhou H.Y., Chen W.J., Wang H., Li Z.B. (2017). Time and frequency characteristics of very fast transient overvoltage in ultra high voltage substation. IEEE Trans. Dielectr. Electr. Insul..

[21] Wang L., Zhang W., Tan X., Chen W., Liang S., Suo C. (2020). Research and experiments on an external miniaturized VFTO measurement system. Sensors.

[22] IEC (2018). Part 102: Alternating Current Disconnectors and Earthing Switches, 2.0. High-Voltage Switchgear and Control Gear.

[23] Wang S., Guo Z., Zhu T., Feng H.K., Wang S.H. (2018). New multi-conductor transmission line model of transformer winding for frequency response analysis considering the frequency-dependent property of the lamination core. Energies.

[24] Liang G.S., Zhang X.L., Wang X.H., Dong H.Y., Cui X. (2006). Research on transformer winding high frequency circuit model under very fast transient overvoltage. Proc. CSEE.

[25] Beura C.P., Beltle M., Tenbohlen S. (2020). Study of the influence of winding and sensor design on ultra-high frequency partial discharge signals in power transformers. Sensors.

[26] Ke X.Z., Li Z.H. (2020). Simulation research on coupling VFTO signal on transformer core grounding wire. High Volt. Appar..

[27] Zhou L., Liao Y.F., Luo B., Zhao Z.Y., Yao C.G. (2017). A simulation study on the frequency response of power transformer winding deformation based on finite element method. Electr. Power Autom. Equip..

[28] Kulkarni S.V., Khaparde S.A. (2016). Transformer Engineering: Design, Technology, and Diagnostics.

